# A triplex real-time PCR method to detect African swine fever virus gene-deleted and wild type strains

**DOI:** 10.3389/fvets.2022.943099

**Published:** 2022-09-15

**Authors:** Hao Yang, Zhong Peng, Wenbo Song, Chen Zhang, Jie Fan, Hongjian Chen, Lin Hua, Jie Pei, Xibiao Tang, Huanchun Chen, Bin Wu

**Affiliations:** ^1^State Key Laboratory of Agricultural Microbiology, College of Veterinary Medicine, Huazhong Agricultural University, Wuhan, China; ^2^Diagnostic Center for Animal Diseases, The Cooperative Innovation Center for Sustainable Pig Production, Wuhan, China; ^3^Hubei Provincial Center for Animal Disease Prevention and Control, Wuhan, China

**Keywords:** B646L, CD2v, MGF_360-14L, African swine fever virus, triplex real-time PCR method, gene-deleted and wild type strains

## Abstract

Currently there is still no effective vaccines and drugs available for African swine fever virus (ASFV), a life-threatening virus to domestic pigs and wild boars. Therefore, accurate diagnosis is important for the prevention and control of the virus. In this study, we developed a triplex real-time PCR method to detect and differentiate ASFV gene-deleted and wild type strains based on three viral genes B646L, MGF_360-14L gene, and CD2v. Standard curves plotted showed that there was a strong linear correlation (*R*^2^ > 0.99) between *Ct* values and the corresponding copy numbers of synthesized standard plasmids. The detection limits of the method for B646L, MGF_360-14L, and CD2v were 78.9, 47.0, and 82.1 copies/μl, respectively. Detection results of different types of swine viruses showed that the method only gave amplification curves to ASFV. Finally, we found the triplex real-time PCR method developed in this study displayed better results on detecting the laboratory sample mocks, and it could be used as a supplemental method to detect ASFV genotype I strains. These findings suggest that the triplex real-time PCR method developed in this study have good specificity and sensitivity. This triplex real-time PCR method might also represent an effective tool for the detection of ASFV gene-deleted and wild type strains.

## Introduction

Since its report for the first time in Kenya in Africa in 1914 (1), African swine fever (ASF) has been a life-threatening disease to global domestic pigs and wild boars with up to 100% case mortality rate (2). ASF is caused by a double-stranded DNA virus belonging to the *Asfarviridae* family, called African swine fever virus (ASFV), which possesses a genome containing ~150–167 protein encoding genes for virus replication and pathogenesis (3). Among these genes, the capsid protein P72 encoding gene B646L is a conserved region for all ASFV strains (including the wild-type and gene-deleted vaccines) and is a common genetic marker for the virus genotyping (4, 5). Based on this gene, ASF strains are divided into 24 different genotypes (genotypes I to XXIV) (4). For the other genes, CD2v and the multigene family (MGF) 360-505R (including MGF_360-14L), are important virulence-associated genes and their deleting strains have become one of the most promising ASF attenuated vaccine candidates (6–10).

In August 2018, the first case of ASF outbreak in China was reported (11). Just <1 year passed by, ASF has been spread in almost all parts of this largest pork producer of the world. Initially, only ASFV genotype II wild-type strains have been isolated in China (11, 12). However, a recent study has found heterogeneous types of ASFV strains, including those with mutations, deletions, insertions, or short-fragment replacement compared with the earliest isolate (Pig/HLJ/2018) in China (12). Compared to the pigs infected by wild-type strains, pigs infected with ASFV variant strains display a prolonged incubation period and mild manifestations; meanwhile, there is a lower amount of detoxification in ASFV variant strain infected pigs than in wild type strain infected pigs; viral strains are always detoxified intermittently in those pigs infected with ASFV variant strains; these characteristics make the ASFV variant strains more difficult to be detected than the wild type strains (13). More seriously, the isolation of two ASFV genotype I field strains (HeN-ZZ-P1-21 and SD/DY-I-21) were reported in 2021, and these two Chinese genotype I isolates lack 10 open reading frames (ORFs), including the MGF_110, MGF_360 and MGF_505 families, compared to the genome sequences of the highly pathogenic genotype I strains L60 and Benin 97/1 (14). These findings suggest a worrisome and complex condition of ASFV prevalence in pig industry in China. Considering there are currently no effective vaccines and/or drugs available, accurate diagnosis is important for the prevention and control of the disease (15). Since real-time PCR method is one of the most-commonly used method and also the recommended method for ASF detection in China (16), we explored the possibility of developing a triplex real-time PCR method targeting the CD2v and MGF_360-14L genes together with B646L for the detection and differentiation of ASFV gene-deleted and wild type strains in this study.

## Materials and methods

### Analysis on the genome sequences of ASFV isolates from China

A total of 14 complete genome sequences of ASFV isolates from China were downloaded from GenBank (https://www.ncbi.nlm.nih.gov/genome/browse/#!/viruses/10302/). Apart from two belonged to ASFV genotype I isolates (HeN/ZZ-P1/2021, Gen-Bank accession no. MZ945536; SD/DY-I/2021, GenBank accession no. MZ945537) (14), the remaining sequences belonged to the genotype II isolates (Supplementary Table S1). Sequence alignments were performed and visualized using BLAST Ring Image Generator (BRIG) (17).

### Standard plasmid construction, primer- and probe-design

Primers and probes targeting B646L, CD2v, and MGF_360-14L were designed using the SnapGene software (version 5.3; https://www.snapgene.com/) and Primer Premier 5 program (18). The probe for B646L was labeled with the 5'-reported dye 6-carboxyfluorescein (FAM) and the 3'-quencher BHQ1, the probe for MGF_360-14L was labeled with the 5'-reported dye Cy5 and the 3'-quencher BHQ2, and the probe for CD2v was labeled with the dye VIC and the 3'-quencher BHQ1 (Table 1). The partial length of B646L (616 bp, base pairs 1,026–1,641), MGF_360-14L gene (541 bp, base pairs 54–594), and CD2v gene (559 bp, base pairs 525–1,083) from the whole genome sequence of ASFV genotype II strain Pig/HLJ/2018 (GenBank accession no. MK333180) were synthesized and cloned into the pUC57 plasmid to generate the recombinant standard plasmids ASFV-B646L-pUC57 (280.70 ng/μl; 7.89 × 10^10^ copies/μl), ASFV-MGF_360-14L-pUC57 (163.34 ng/μl; 4.70 × 10^10^ copies/μl), and ASFV-CD2v-pUC57 (288.03 ng/μl; 8.21 × 10^10^ copies/μl), respectively. A recombinant standard plasmid pUC57-I-B646L-CD2v-MGF_360-14L (100.00 ng/μl) carrying B646L, CD2v, MGF_360-14L from the genotype I strain Benin 97/1 (GenBank accession no. AM712239) was also synthesized.

**Table 1:** Primers and probe sequences used in this study.

**Primer/probe**	**Sequence (5'-3')**	**Targe gene**	**Size (bp)**
P72-F	CTACCTGGAACATCTCCGATCA	B646L	106
P72-R	CTTATCTCTGCGTGGTGAGT		
P72-P	6-FAM-CTCATCAACACCGAGATTGGCACAAG-BHQ-1		
MGF-F	TTGGGGCGCAAATCCTGAAT	MGF_360-14L	86
MGF-R	GCGTTAAGCCTCCCAGTTC		
MGF-P	Cy5-ACACAGCCGCTTTAGATACACGGCA-BHQ-2		
CD2v-F	CCACCACCTGAATCTAATGAAGAAG	CD2v	111
CD2v-R	CTGATAACGACTGTAAGGCTTAGG		
CD2v-P	VIC-ACAATGTCAGCATGATGACACCACTTCC-BHQ-1		

### PCR reaction volume and optimization of amplification conditions

Genomic DNA was extracted using a Vazyme DNA/RNA Extraction Kit (Cat NO. RM-201-02; Nanjing, China) following the manufactory instructions. The triplex real-time PCR assay was performed in a 25-μl reaction volume, which contains template DNA 5-μl, AceQ^®^ Uniwersal U+ Probe Master Mix (Vazyme, Nanjing, China) 12.5-μl, each of the forward and reverse primers (0.12, 0.16, 0.20, 0.24, 0.28, or 0.32 μM), each of the TaqMan probes (0.12, 0.16, 0.20, 0.24, 0.28, or 0.32 μM), and nuclease-free water up to 25-μl. PCR assay was performed on an CFX96 Touch Real-Time PCR Detection System (Bio-Rad, Hercules, CA) with the following conditions: 95°C for 5 min, followed by 40 cycles of 95°C for 15 s, annealing at different temperatures (55–60°C) for 45 s. Fluorescence was recorded at 59°C. Copy number was calculated using the formula (Copy number = [(6.02 × 10^23^) × ([ng/μl] × 10^−9^)]/[DNA length × 660]) described previously (19). In addition, plasmids (ASFV-B646L-pUC57, ASFV-MGF_360-14L-pUC57, and ASFV-CD2v-pUC57) with a series of 10-fold dilution (10^−2^-10^−11^) were used as the templates to validate the method.

### Construction of standard curves

To generate standard curves, a series of 10-fold dilutions (10^−2^-10^−9^) were given to the three synthesized standard plasmids (ASFV- B646L-pUC57, ASFV-MGF_360-14L-pUC57, and ASFV-CD2v-pUC57), which were used as the template DNA to perform the triplex real-time PCR assays. Standard curves were generated based on the cycle threshold (*Ct*) values and the copy numbers (lg values) of the template DNA. Coefficients of determination (*R*^2^) were calculated using GraphPad Prism v. 8.0.1 (https://www.graphpad.com/scientific-software/prism/).

### Validation of specificity and sensitivity

To test the stability of the generated triplex real-time PCR method, separated assays were performed to detect the recombinant standard plasmids at different concentrations and compared the Ct values. The specificity and sensitivity of the generated triplex real-time PCR method was validated using the genomic DNA extracted from the other viruses, including pseudorabies virus (PRV), porcine reproductive and respiratory syndrome virus (PRRSV), Japanese encephalitis virus (JEV), porcine parvovirus (PPV), and porcine circovirus type 2 (PCV2). In addition, the synthesized plasmids pUC57-ΔMGF_360-14L, pUC57-ΔCD2v, pUC57-ΔMGF_360-14L/CD2v, and pUC57-MGF_360-14L/CD2v were also used as the template DNA to validate the method. We also compared the detection results of the triplex real-time PCR method developed in this study (hereinafter referred to as the “tr-PCR”) to those of a reported triplex real-time PCR method (hereinafter referred to as the “r-PCR”) (5), and a recommended real-time PCR method in China (hereinafter referred to as the “gb-PCR”) (16). DNA extracted from different types of samples (soil, water, pig anticoagulant blood, pig feces, environmental swabs, pig tissues) mixed ASFV genomic DNA (extracted from positive field samples) were detected by the three real-time PCR methods.

### Evaluation of the possible application of the triplex real-time PCR method to detect ASFV genotype I strains

To assess the possible application of the triplex real-time PCR method to detect ASFV genotype I strains, the synthesized plasmid pUC57-I-B646L-CD2v-MGF_360-14L (10^5^ copy/μl) was used as the template for PCR detection. The triplex real-time PCR assay was performed in a 25-μl reaction volume, which contains template DNA 5-μl, AceQ^®^ Uniwersal U+ Probe Master Mix (Vazyme, Nanjing, China) 12.5-μl, each of the forward and reverse primers (0.20 μM), each of the TaqMan probes (0.20 μM), and nuclease-free water up to 25-μl. PCR assay was performed on an CFX96 Touch Real-Time PCR Detection System (Bio-Rad, Hercules, CA) with the following conditions: 95°C for 5 min, followed by 40 cycles of 95°C for 15 s, annealing at different temperatures (59°C) for 45 s.

### Specific statements

The inactivation of an ASFV strain used for the simulation of virus-containing samples was performed in Huazhong Agricultural University Animal Biosafety Level-3 Laboratory (ABSL-3, <city>Wuhan</city>, China) following the requirements of the Ministry of Agriculture and Rural Affairs (MARA) of the People's Republic of China (MARA General Office Document no. [2019] 12).

## Results

### Sequence analysis indicates the condition of application

**Figure 1:**
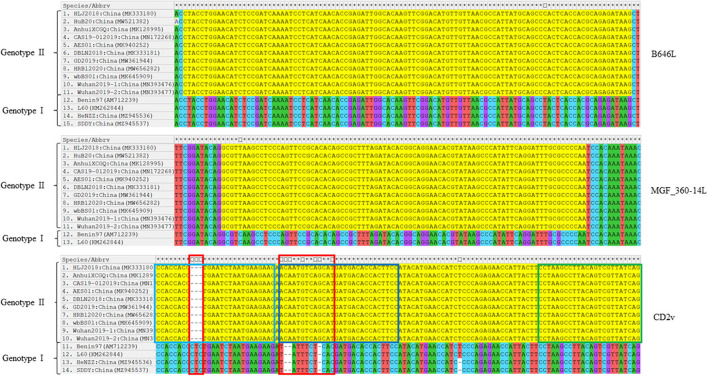
Nucleotide sequence comparisons of the primer-target regions of B646L, CD2v, and MGF_360-14L from different ASFV strains. The primer-target regions of B646L, CD2v, and MGF_360-14L are heighted in yellow. In CD2v gene, different regions between the genotype I strains and genotype II strains are shown in red boxes; CD2v forward primer, probe, and reverse primer are shown in light blue, dark blue, and green boxes.

Sequence comparisons of the primer-target regions of B646L, CD2v, and MGF_360-14L from different ASFV strains demonstrated that B646L was highly conserved, not only between ASFV strains belonging to the same genotype, but also between the genotype I and genotype II strains (Figure 1). Although MGF_360-14L was conserved among the eleven Chinese genotype II strains and the two genotype I strains L60 and Benin 97/1, this gene was missing in the genome sequences of the two genotype I strains HeNZZ (GenBank accession no. MZ945536) and SDDY (GenBank accession no. MZ945537) from China (Figure 1; Supplementary Figure S1). For CD2v, sequence alignments revealed that this gene was conserved among the Chinese genotype II strains, but it was various between the Chinese genotype II strains and the genotype I strains (Figure 1). Based on the above findings, it may conclude that the primer-target area of B646L in this study was a proper marker for the detection of presence of ASFV; while the primer-target area of MGF_360-14L could be used to differentiated MGF_360-14L-deletion strains from the wild type strains; and the primer-target area of CD2v was applicable for differentiating CD2v-deletion strains from the wild type strains, or differentiating genotype II wild type strains from genotype I wild type strains.

### Optimization of the amplification conditions

We next investigated the optimal concentrations of the primers and probes. To achieve this, we detected 10^4^ copies/μl of plasmids with different concentrations of primers and probes (0.12, 0.16, 0.20, 0.24, 0.28, or 0.32 μM) at the annealing temperature of 59.0°C. The results revealed that an optimal amplification condition occurred when the concentrations were set as 0.20 μM (Supplementary Figure S2A). To explore the optimal annealing temperature for tr-PCR, we detected the plasmids with 0.20 μM of primers and probes at 55.0, 56.0, 57.0 58.0, 59.0, and 60.0°C. The results revealed that the tr-PCR assay displayed the optimal amplification conditions at the annealing temperature of 59.0°C (Supplementary Figure S2B).

### Detection limit and standard curves

**Figure 2:**
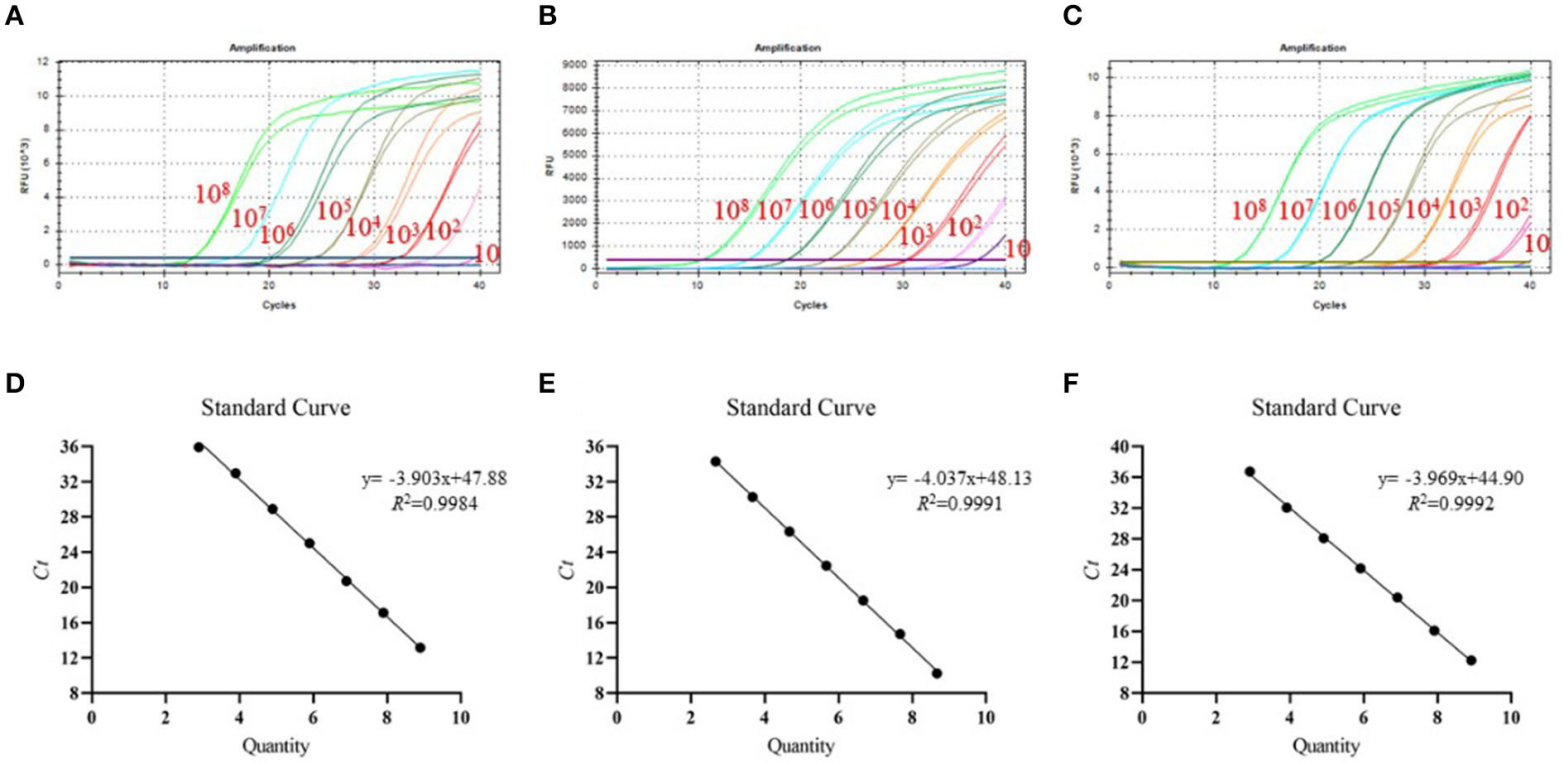
Detection limit and standard curves of the triplex real-time PCR method developed in this study. **(A)** Detection limit for B646L; **(B)** Detection limit for MGF_360-14L; **(C)** Detection limit for CD2v; **(D)** Plasmid DNA standard curve for B646L, *y* = −3.903x + 47.88, *R*^2^ = 0.9984; **(E)** Plasmid DNA standard curve for MGF_360-14L, y = −4.037x + 48.13, *R*^2^ = 0.9991; **(F)** Plasmid DNA standard curve for CD2v gene, y = −3.969x + 44.90, *R*^2^ = 0.9992.

To test the detection limit of tr-PCR, a series of 10-fold dilutions were given to ASFV-B646L-pUC57 (from 7.89 × 10^10^ to 78.9 copies/μl), ASFV-MGF_360-14L-pUC57 (from 4.70 × 10^10^ to 47.0 copies/μl), and ASFV-CD2v-pUC57 (from 8.21 × 10^10^ to 82.1 copies/μl). Plasmids with different copy numbers were then detected using tr-PCR. The results revealed that the detection limits for B646L, MGF_360-14L, and CD2v were 78.9, 47.0, and 82.1 copies/μl, respectively (Figures 2A–C). Standard curves plotted using GraphPad Prism software v. 8.0.1 showed that there was a strong linear correlation (*R*^2^ > 0.99) between Ct values and the corresponding copy numbers of ASFV- B646L-pUC57, ASFV-MGF_360-14L-pUC57, and ASFV-CD2v-pUC57. The standard curves of the three standard plasmids were plotted with slopes of −3.903, −4.037, and −3.969, respectively (Figures 2D–F).

### Stability, specificity, and sensitivity of the triplex real-time PCR method

**Figure 3:**
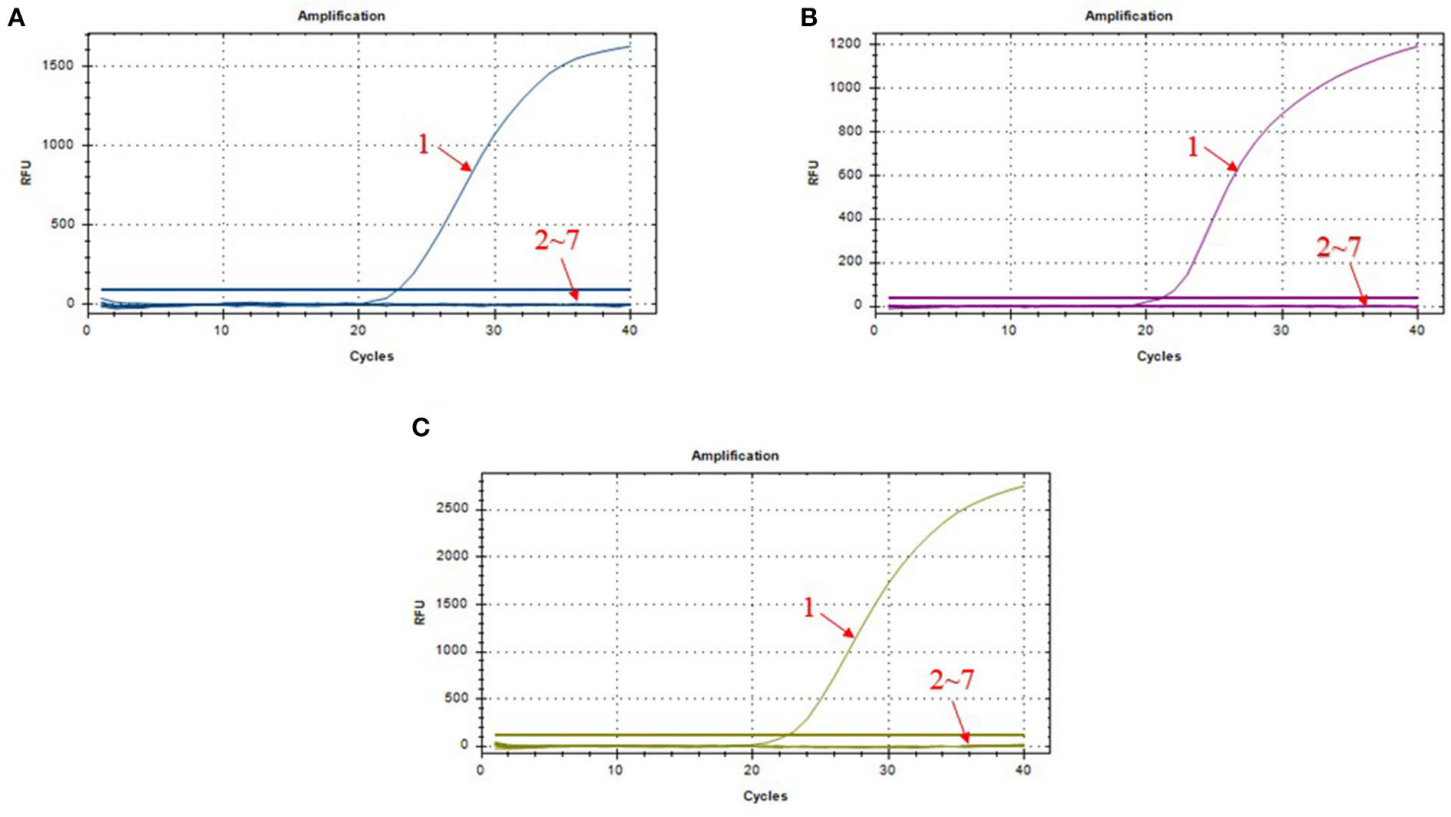
Amplification curves in the specificity test of triplex real-time PCR assay. **(A)** amplification curves of B646L; **(B)** amplification curves of MGF_360-14L; **(C)** amplification curves of CD2v; 1: ASFV; 2–7: PRV, PRRSV, JEV, PPV, PCV2, and DNA-free water.

We chose different standard plasmids at ~10^2^ copies/μl or ~10^6^ copies/μl as the DNA templates to test the coefficient of variation (*C.V*.) values of the method. The results showed that *C.V*. values determined within different detection groups and between different detection groups were lower than 1.5% (Table 2), indicating the developed method possesses a good stability. Specificity tests revealed that only DNA samples from ASFV showed positive amplification curves for the three fluorescence channels of FAM, Cy5 and VIC; while those from PRV, PRRSV, JEV, PPV, and PCV2 did not show amplification curves (Figures 3A–C).

**Table 2:** Validation of the Detection repeatability of the developed triplex real-time PCR method.

**Genes**	**DNA (copies/μL)**	**Rounds of testing**	***Ct* mean^a^**	***Ct* Standard deviation**	***C*.*V*.^a^**	***C.V*. between groups**
B646L	7.89 × 10^2^	1	33.34	0.24	0.73%	0.74%
		2	32.99	0.26	0.79%	
		3	33.47	0.42	1.25%	
	7.89 × 10^3^	1	29.53	0.11	0.37%	0.49%
		2	29.33	0.16	0.54%	
		3	29.60	0.21	0.70%	
	7.89 × 10^4^	1	26.68	0.09	0.33%	0.17%
		2	26.59	0.08	0.29%	
		3	26.63	0.05	0.19%	
	7.89 × 10^5^	1	23.14	0.12	0.54%	0.19%
		2	23.16	0.09	0.40%	
		3	23.07	0.02	0.11%	
	7.89 × 10^6^	1	19.15	0.13	0.67%	0.54%
		2	18.95	0.15	0.77%	
		3	19.02	0.09	0.46%	
MGF_360-14L	4.70 × 10^2^	1	35.29	0.66	1.86%	1.35%
		2	34.41	1.40	4.06%	
		3	34.58	0.20	0.56%	
	4.70 × 10^3^	1	30.53	0.06	0.18%	0.36%
		2	30.62	0.05	0.16%	
		3	30.41	0.09	0.30%	
	4.70 × 10^4^	1	26.06	0.19	0.71%	0.28%
		2	25.96	0.23	0.90%	
		3	26.10	0.06	0.24%	
	4.70 × 10^5^	1	22.46	0.04	0.16%	0.56%
		2	22.49	0.09	0.41%	
		3	22.69	0.18	0.78%	
	4.70 × 10^6^	1	18.49	0.05	0.28%	0.14%
		2	18.44	0.13	0.72%	
		3	18.48	0.05	0.28%	
CD2v	8.21 × 10^2^	1	32.18	0.16	0.51%	0.29%
		2	32.03	0.20	0.63%	
		3	32.01	0.36	1.11%	
	8.21 × 10^3^	1	29.40	0.07	0.25%	0.31%
		2	29.39	0.05	0.17%	
		3	29.55	0.11	0.36%	
	8.21 × 10^4^	1	26.48	0.17	0.64%	0.23%
		2	26.43	0.30	1.15%	
		3	26.36	0.42	1.61%	
	8.21 × 10^5^	1	23.78	0.06	0.27%	0.01%
		2	23.78	0.04	0.19%	
		3	23.78	0.06	0.25%	
	8.21 × 10^6^	1	19.76	0.07	0.35%	0.18%
		2	19.69	0.02	0.10%	
		3	19.75	0.10	0.53%	

a Ct, cycle threshold; C.V., coefficient of variation.

**Figure 4:**
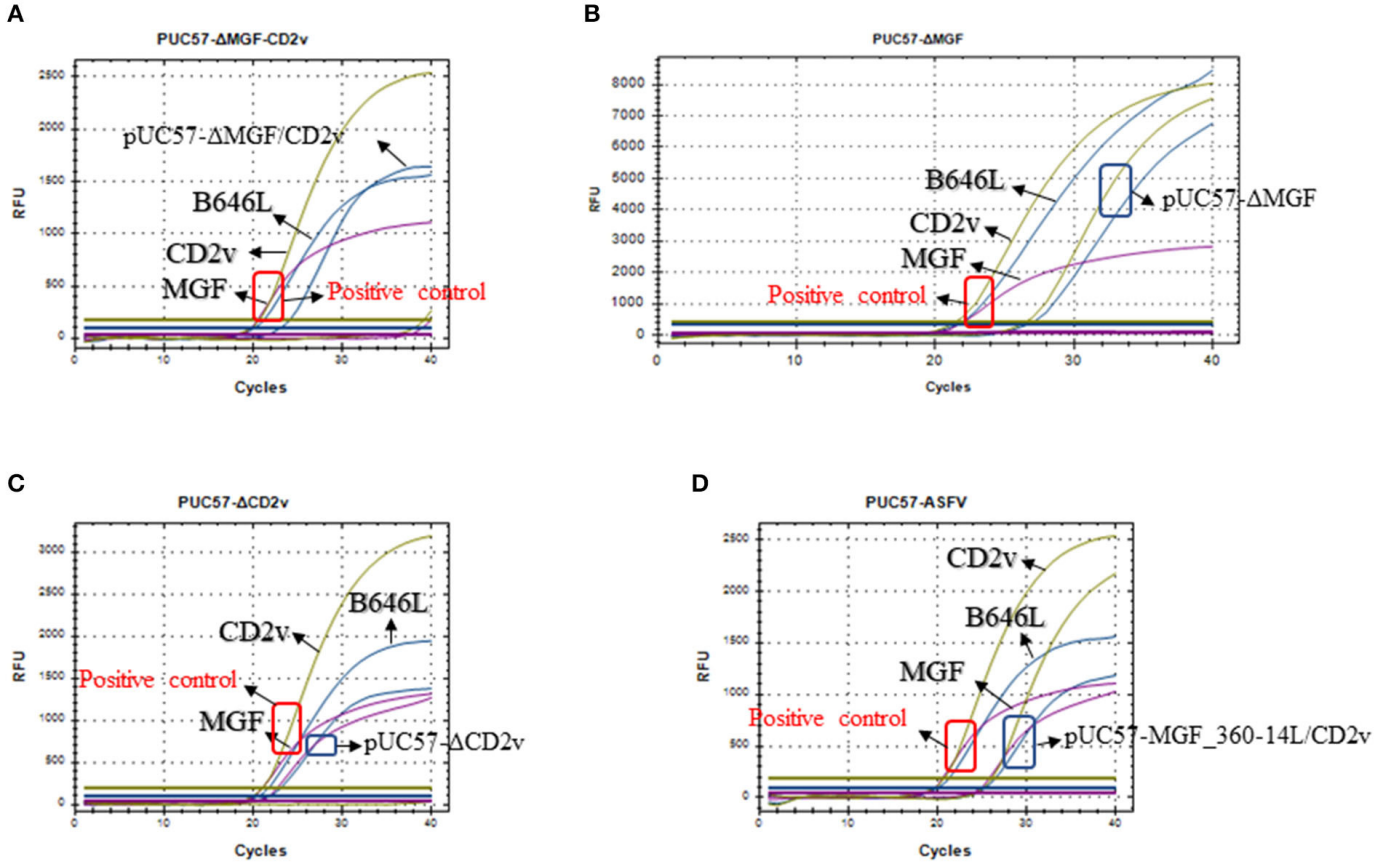
Detection of the triplex real-time PCR methods developed in this study on the mocks with different gene deletions. **(A)** Detection of the MGF_360-14L/CD2v double deletion plasmid pUC57-ΔMGF_360-14L/CD2v; **(B)** Detection of the MGF_360-14L deletion plasmid pUC57-ΔMGF_360-14L; **(C)** Detection of the CD2v deletion plasmid pUC57-ΔCD2v; **(D)** Detection of the wild type plasmid pUC57-MGF_360-14L/CD2v; curves in blue represents the amplification curves of B646L; curves in purple represents the amplification curves of MGF_360-14L; curves in glode represents the amplification curves of CD2v. In all panels, a plasmid containing all three genes was used a positive control template gene and the amplification results are shown in red box; while the detection results based on different types of plasmids are shown in blue box.

Next, we used the synthesized plasmids pUC57-ΔMGF_360-14L, pUC57-ΔCD2v, pUC57-ΔMGF_360-14L/CD2v, and pUC57-MGF_360-14L/CD2v as the template DNA to validate the method. The results revealed that the method could differentiate the three types of gene-deletion plasmids as well as the gene-completeness plasmid, suggesting the method is able to differentiate ASFV wild type and gene-deletion strains (Figure 4).

### Comparison of the detection results of different real-time PCR methods

**Figure 5:**
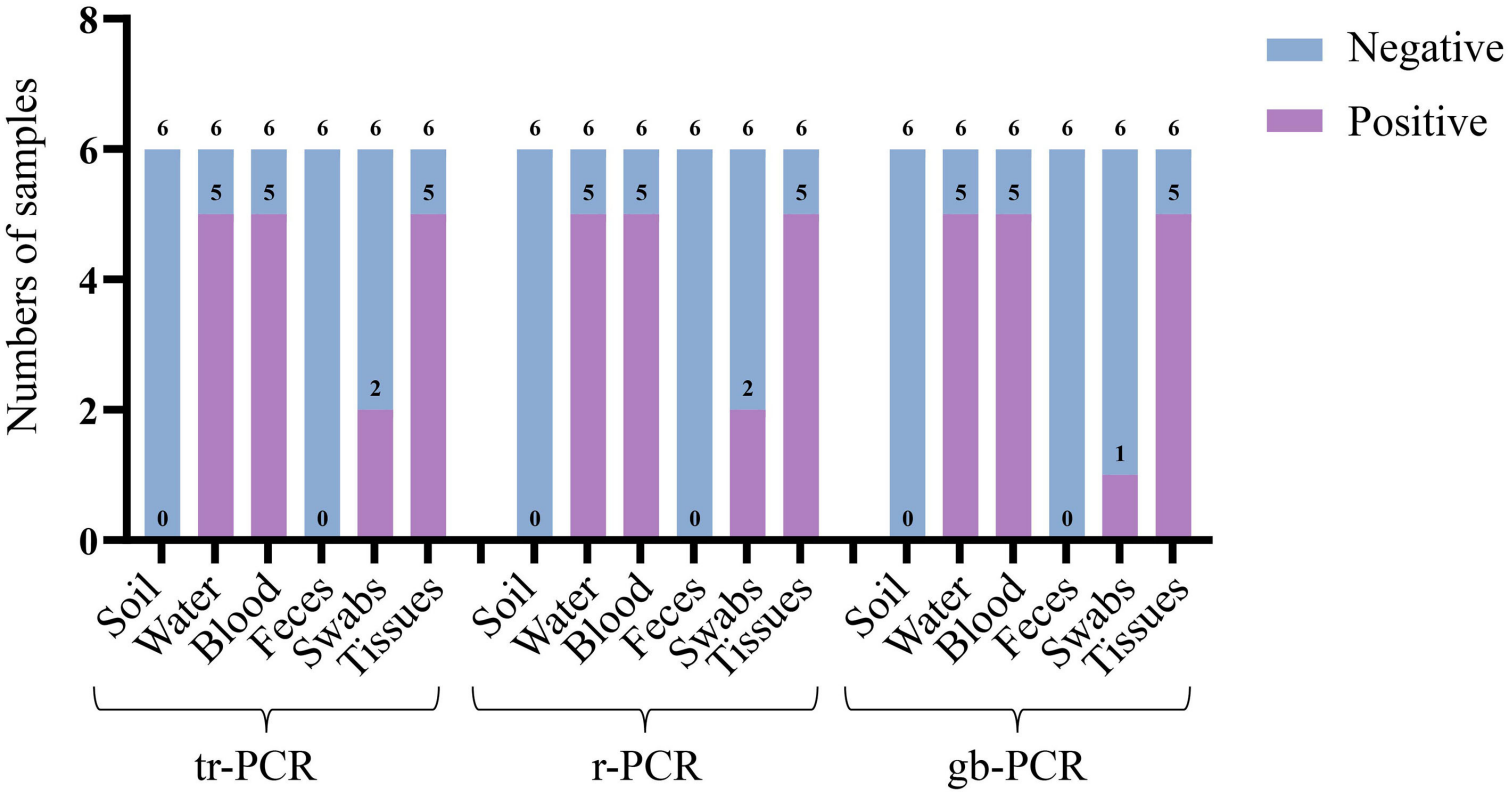
Detection of different real-time PCR methods on different laboratory sample mocks.

To evaluate the accuracy of tr-PCR, inactivated ASFV strains were mixed with soil samples (*n* = 6), water samples (*n* = 6), pig anticoagulant blood samples (*n* = 6), pig fecal samples (*n* = 6), table and floor swabs (spraying on the surfaces of tables and/or floors then collecting the swabs), and/or pig tissue samples (*n* = 6). Genomic DNA were extracted from these samples and were detected using tr-PCR, r-PCR (5), and gb-PCR (16). The results revealed that 17 samples, 17 samples, and 16 samples were detected to be positive by using these three methods, respectively, indicating that tr-PCR showed a similar result of detection to r-PCR (Figure 5; Supplementary Table S2).

### The possible application of the triplex real-time PCR method to detect ASFV genotype I strains

**Figure 6:**
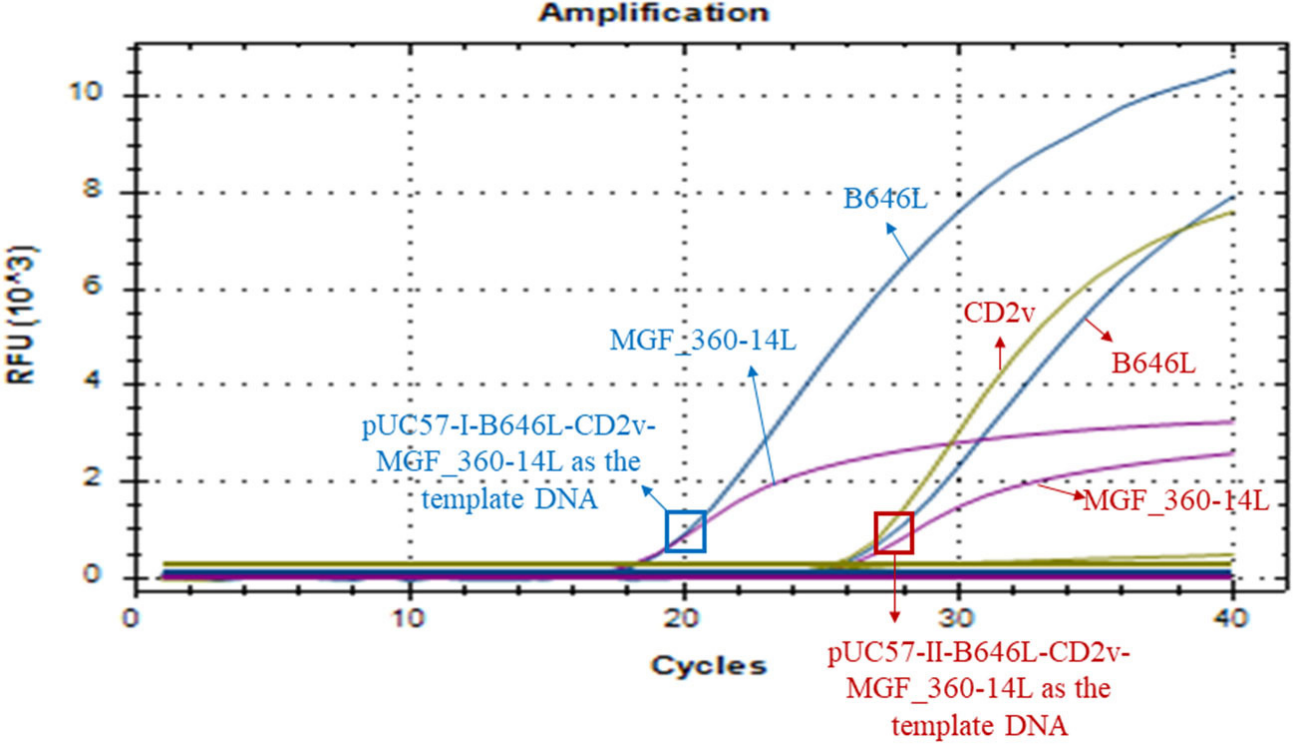
Amplification curves of triplex real-time PCR assay on detecting B646L, CD2v, and MGF_360-14L from ASFV genotype I strains. The result of the method using a plasmid containing B646L, CD2v, and MGF_360-14L (pUC57-I-B646L-CD2v-MGF_360-14L) from ASFV genotype I strains is shown in blue, while result of the method using a plasmid containing B646L, CD2v, and MGF_360-14L (pUC57-II-B646L-CD2v-MGF_360-14L) from ASFV genotype II strains is shown in red.

Our above sequence alignment results revealed that the genotype I strains reported in China (HeNZZ and SDDY) lacked the MGF_360-14L gene, and the CD2v gene was different between the genotype I and genotype II strains (Figure 1; Supplementary Figure S1). Therefore, the triplex real-time PCR method developed in this study might have a possible use in detecting ASFV strains. To explore this, the synthesized plasmid pUC57-I-B646L-CD2v-MGF_360-14L was used as the template to perform the PCR assays. The results revealed that the triplex real-time PCR method was able to give amplifying curves of the B646L and MGF_360-14L of ASFV genotype I strains but it did not give the amplifying curve of CD2v (Figure 6).

## Discussion

ASF is a World Organization for Animal Health (WOAH) listed animal infectious disease and one of the most severe threats to global pig industry. Despite of ~107 years of research, there is still no effective vaccines and/or drugs available for the treatment of the disease (20). Therefore, accurate detection is important for the control and prevention of ASF (15, 21). Since real-time PCR method is one of the most-commonly used method and also the recommended method for ASF detection in China (16), we therefore constructed a triplex real-time PCR method in this study.

The triplex real-time PCR method in this study was developed based on three genes B646L, CD2v (EP402R), and MGF_360-14L. Among them, B646L is a conserved gene among different ASFV strains and is commonly used for the detection and genotyping of ASFV (5, 16, 22, 23). According to the published data, only ASFV genotype II and genotype I strains have been reported in China until recently (12–14), and genotype II is the epidemic genotype in the field (12, 24, 25). The three genes we selected for developing the real-time PCR method were conserved among the wild type. Particularly, recent studies have reported the prevalence of CD2v/MGF360-deleted ASFV strains in pig farms in China (12, 14). Therefore, the triplex real-time PCR method developed based on these three genes might have a potential use for the detection of ASFV gene deletion strains and wild type strains. It is worthy of note that both CD2v (EP402R) and MGF_360-14L are important genes most-frequently deleted for vaccine study (6–10). While no approved commercial vaccine is currently available to protect pigs from the virus in China, the triplex real-time PCR method may also represent a potential choice to differentiate ASFV vaccines strains and wild type strains in the future if there are associated vaccines approved. However, considering many other genes, e.g., A137R (26), I177L (27), E184L (28), etc., have been also demonstrated as suitable targets for deletion to develop vaccine candidates, these genes should be also included for the development of proper multiplex real-time PCR methods for the differentiation of ASFV vaccines strains and wild type strains in future.

Our whole genome sequence alignments also found B646L and CD2v (EP402R) were relatively conserved between ASFV genotype II and genotype I strains, but the two genotype I strains recently isolated from China lacked the MGF_360-14L gene. These findings are in agreement with the recent study reporting the isolation of these two genotype I strains (14). Therefore, the triplex real-time PCR method developed in this study may also have a potential use to detect the recent emerging genotype I strains in China.

Specificity and sensitivity are important measures of the diagnostic accuracy of a test (29). In this study, we conducted different assays to validate the specificity and sensitivity of the developed triplex real-time PCR method. Detection results of different types of swine viruses (ASFV, PRV, PRRSV, JEV, PPV, and PCV2) showed that the method only gave amplification curves to ASFV, and the detection limits of the for B646L, MGF_360-14L, and CD2v were 78.9, 47.0, and 82.1 copies/μl, respectively. These results are in agreement with the recent reported triplex real-time PCR method (5). Moreover, the triplex real-time PCR method developed in this study displayed better results on detecting the laboratory sample mocks. These findings suggest that the triplex real-time PCR method developed in this study have good specificity and sensitivity.

A noteworthy point is that a target gene of the triplex real-time PCR method developed in this study is MGF_360-14L. While ASFV genotype II strains lacking this gene has been reported in China (12), ASFV genotype I strains (HeNZZ and SDDY) reported in China also do not contain this gene (14). In this regard, the triplex real-time PCR method developed in this study could either detect ASFV genotype II MGF_360-14L-deletion strains or detect ASFV genotype I strains. In addition, the triplex real-time PCR method developed in this study did not give the amplifying curve of the CD2v gene of ASFV genotype I strains. Considering a few cases report the isolation of ASFV genotype I strains, the triplex real-time PCR method developed in this study could be used as a supplemental method to detect ASFV genotype I strains.

In summary, we developed a triplex real-time PCR method to detect ASFV gene-deleted and wild type strains. This method was found to be specific and sensitive, and it exhibited better results on detecting both laboratory sample mocks than the other used real-time PCR methods. Most importantly, the triplex real-time PCR method also demonstrated a potential to initially detect ASFV genotype I strains. It might also represent an effective tool for the detection of ASFV gene-deleted and wild type strains.

## Data availability statement

The original contributions presented in the study are included in the article/Supplementary material, further inquiries can be directed to the corresponding author/s.

## Author contributions

HY, ZP, and BW: conceptualization. HY, WS, CZ, JF, HoC, LH, JP, and XT: methodology, formal analysis, and investigation. HY and ZP: writing—original draft preparation. HuC, ZP, and BW: writing—review and editing. BW: funding acquisition. ZP and BW: supervision. All authors contributed to the article and approved the submitted version.

## Funding

This work was supported in part by the National Natural Science Foundation of China (grant no. U20A2059) and Hubei Provincial Key Research and Development Program (grant no. 2021BBA085).

## Conflict of interest

The authors declare that the research was conducted in the absence of any commercial or financial relationships that could be construed as a potential conflict of interest.

## Publisher's note

All claims expressed in this article are solely those of the authors and do not necessarily represent those of their affiliated organizations, or those of the publisher, the editors and the reviewers. Any product that may be evaluated in this article, or claim that may be made by its manufacturer, is not guaranteed or endorsed by the publisher.

## Supplementary material

Supplementary Figure S1 Sequence comparison of ASFV isolates from China.

Supplementary Figure S2 Results of the optimizations of the primer/probe concentrations **(A)** and annealing temperatures **(B)**.

Supplementary Table S1 The genome sequences of ASFV Chinese isolates used in this study.

Supplementary Table S2 Results of three real-time PCR methods detecting the laboratory sample mocks.
