# Supplementary material for: A triplex real-time PCR method to detect African swine fever virus gene-deleted and wild type strains
Source: Front Vet Sci. 2022 Sep 15;9:943099. doi: 10.3389/fvets.2022.943099 (PMC9521421; doi:10.3389/fvets.2022.943099)
Supplement: Supplementary Figure S1 — Sequence comparison of ASFV isolates from China. [file Data_Sheet_1.ZIP › Supplementary materials/Table S1.docx]

**Table S1** The genome sequences of ASFV Chinese isolates used in this study

| **Strain** | **Genotype** | **Year of isolation** | **Place of isolation** | **GenBank accession** |
| --- | --- | --- | --- | --- |
| China2018AnhuiXCGQ | II | 2018 | Anhui, China | MK128995 |
| ChinaCAS19-012019 | II | 2019 | Guangdong, China | MN172368 |
| CN2019lnnerMongolia-AES01 | II | 2019 | lnnerMongolia, China | MK940252 |
| DBLN2018 | II | 2018 | Liaoning, China | MK333181 |
| GD2019 | II | 2019 | Guangdong, China | MW361944 |
| HeilongjiangHRB12020 | II | 2020 | Heilongjiang, China | MW656282 |
| HLJ2018 | II | 2018 | Heilongjiang, China | MK333180 |
| HuB20 | II | 2020 | Hubei, China | MW521382 |
| SY-18 | II | 2018 | Liaoning, China | MH766894 |
| wbBS01 | II | 2018 | China | MK645909 |
| Wuhan 2019-1 | II | 2019 | Hubei, China | MN393476 |
| Wuhan2019-2 | II | 2019 | Hubei, China | MN393477 |
| HeN/ZZ-P1/2021 | I | 2021 | Henan, China | MZ945536 |
| SD/DY-I/2021 | I | 2021 | Shandong, China | MZ945537 |
