# Supplementary material for: A triplex real-time PCR method to detect African swine fever virus gene-deleted and wild type strains
Source: Front Vet Sci. 2022 Sep 15;9:943099. doi: 10.3389/fvets.2022.943099 (PMC9521421; doi:10.3389/fvets.2022.943099)
Supplement: Supplementary Figure S1 — Sequence comparison of ASFV isolates from China. [file Data_Sheet_1.ZIP › Supplementary materials/Table S2.docx]

**Table S2** Results of three real-time PCR methods detecting the laboratory sample mocks

| **Numbers** | **Types** | **gb-PCR^a^** | **r-PCR^b^** | **tr-PCR^c^** | **Numbers** | **Types** | **gb-PCR^a^** | **r-PCR^b^** | **tr-PCR^c^** |
| --- | --- | --- | --- | --- | --- | --- | --- | --- | --- |
| 1 | Water | + | + | + | 21 | Soil | - | - | - |
| 2 | Water | + | + | + | 22 | Soil | - | - | - |
| 3 | Water | + | + | + | 23 | Soil | - | - | - |
| 4 | Water | + | + | + | 24 | Soil | - | - | - |
| 5 | Water | + | + | + | 25 | Pig feces | - | - | - |
| 6 | Water | - | - | - | 26 | Pig feces | - | - | - |
| 7 | Environmental samples | - | - | + | 27 | Pig feces | - | - | - |
| 8 | Environmental samples | - | - | - | 28 | Pig feces | - | - | - |
| 9 | Environmental samples | - | - | - | 29 | Pig feces | - | - | - |
| 10 | Environmental samples | + | + | + | 30 | Pig feces | - | - | - |
| 11 | Environmental samples | - | + | + | 31 | Pig tissue samples | + | + | + |
| 12 | Environmental samples | - | - | - | 32 | Pig tissue samples | + | + | + |
| 13 | Pig  anticoagulant  blood | + | + | + | 33 | Pig tissue samples | + | + | + |
| 14 | Pig  anticoagulant  blood | + | + | + | 34 | Pig tissue samples | + | + | + |
| 15 | Pig  anticoagulant  blood | + | + | + | 35 | Pig tissue samples | + | + | + |
| 16 | Pig  anticoagulant  blood | + | + | + | 36 | Pig tissue samples | - | - | - |
| 17 | Pig  anticoagulant  blood | + | + | + | Positive control | | + | + | + |
| 18 | Pig  anticoagulant  blood | - | - | - | Negative control | | - | - | - |
| 19 | Soil | - | - | - |  |  |  |  |  |
| 20 | Soil | - | - | - |  |  |  |  |  |

^a^ gb-PCR: A national standard real-time PCR for the diagnosis of ASFV (GB/T 18648-2020)

^b^ r-PCR: A reported triplex real-time PCR method (Lin et al., J Virol Methods. 2020 Jun; 280: 113875)

^c^ tr-PCR: The triplex real-time PCR method developed in this study

“+” refers to be positive; “-” refers to be negative
