# Supplementary figures and images for: A triplex real-time PCR method to detect African swine fever virus gene-deleted and wild type strains
Source: Front Vet Sci. 2022 Sep 15;9:943099. doi: 10.3389/fvets.2022.943099 (PMC9521421; doi:10.3389/fvets.2022.943099)

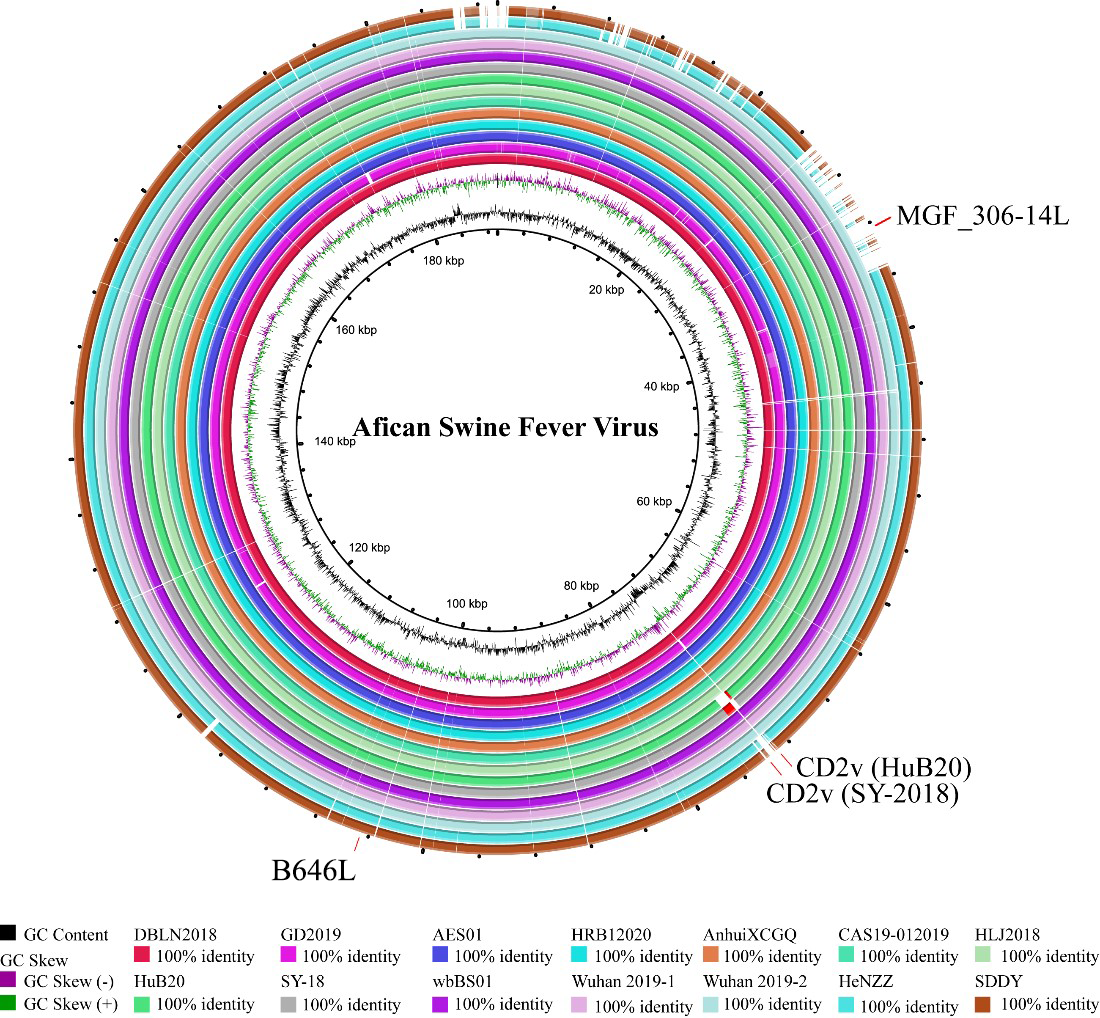

Supplement: Supplementary Figure S1 — Sequence comparison of ASFV isolates from China. [file Data_Sheet_1.ZIP › Supplementary materials/Figure S1.tif]

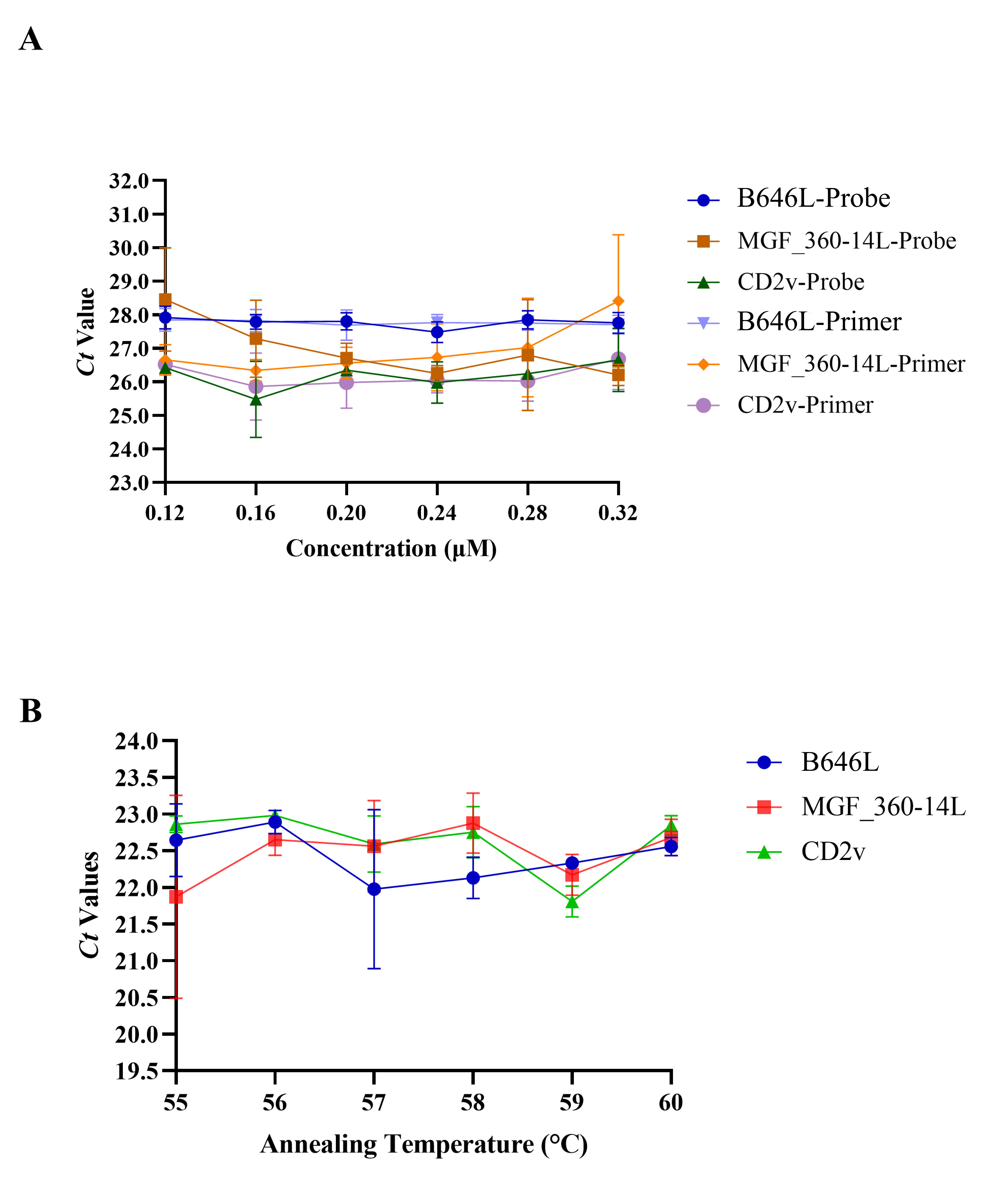

Supplement: Supplementary Figure S1 — Sequence comparison of ASFV isolates from China. [file Data_Sheet_1.ZIP › Supplementary materials/Figure S2.tif]
